# The Pathogenesis of Cardiac Fibrosis: A Review of Recent Progress

**DOI:** 10.3390/ijms23052617

**Published:** 2022-02-27

**Authors:** Kazuaki Maruyama, Kyoko Imanaka-Yoshida

**Affiliations:** Department of Pathology and Matrix Biology, Graduate School of Medicine, Mie University, 2-174 Edobashi, Tsu 514-8507, Japan; imanaka@med.mie-u.ac.jp

**Keywords:** cardiac fibrosis, pathogenesis of cardiac fibrosis, signaling of cardiac fibrosis, marker of cardiac fibroblast

## Abstract

Fibrosis is defined as the excessive deposition of extracellular matrix (ECM) proteins in the interstitium. It is an essential pathological response to chronic inflammation. ECM protein deposition is initially protective and is critical for wound healing and tissue regeneration. However, pathological cardiac remodeling in excessive and continuous tissue damage with subsequent ECM deposition results in a distorted organ architecture and significantly impacts cardiac function. In this review, we summarized and discussed the histologic features of cardiac fibrosis with the signaling factors that control it. We evaluated the origin and characteristic markers of cardiac fibroblasts. We also discussed lymphatic vessels, which have become more important in recent years to improve cardiac fibrosis.

## 1. Introduction

Heart failure (HF) is a complex syndrome resulting from structural and functional impairments of the heart [1]. From the pathological perspective, HF is characterized by interstitial fibrosis, chamber remodeling, and reduced ventricular compliance [2].

The adult mammalian heart has limited regenerative capacity. Thus, repair processes are crucial after injury. They involve inflammatory cell infiltration, the removal of necrotic cardiomyocytes, and the formation of capillary-enriched granulation tissues. Then, fibrotic scars replace granulation tissues to preserve myocardial structural and functional integrity. Fibrosis is defined as the excessive deposition of extracellular matrix (ECM) proteins, mostly collagen fibers, in the interstitium. It is an essential pathological response to chronic inflammation. ECM protein deposition is initially protective and is critical for wound healing and tissue regeneration. The ECM is also important for maintaining physiological conditions. For instance, cardiomyocyte–ECM interactions mediate cellular behaviors through cell surface receptors, which act as signal transducers for cell proliferation, migration, survival, and differentiation [3]. However, pathological cardiac remodeling in excessive and continuous tissue damage with subsequent ECM deposition results in distorted organ architecture and significantly impacts cardiac function [2,4]. Collectively, heart diseases, such as myocardial infarction (MI), cardiac hypertrophy due to pressure or volume overload, diabetic cardiomyopathy, and dilated cardiomyopathy (DCM) [2,5,6,7] are involved in cardiac fibrosis progression. Although fibrosis has pathophysiological importance in cardiovascular disease development, the development and molecular mechanisms of cardiac fibrosis still require investigation.

In this review, we summarized and discussed the histologic features of cardiac fibrosis and the signaling factors that control it. We evaluated the origin and characteristic markers of cardiac fibroblasts (CFs) under normal and pathophysiological conditions. We also discussed lymphatic vessels, which have become more important in recent years to improve cardiac fibrosis.

## 2. Histological Features of Cardiac Fibrosis

Microscopically, myocardial fibrosis is divided into three groups according to the fibrous deposition patterns [8,9]. Replacement or reparative fibrosis is related to cardiomyocyte loss and rearrangement by collagen fibers. A typical example is scar tissue after MI. Interstitial fibrosis is characterized by fibrous deposits surrounding the cardiac muscle bundles (the perimysium) and individual cardiomyocytes (the endomysium), and perivascular fibrosis is characterized by fibrous deposits located in the perivascular space. Interstitial and perivascular fibrosis are sequential pathological lesions. Hence, this is sometimes called reactive fibrosis. Reactive fibrosis in the absence of the massive loss of cardiomyocytes is caused by prolonged activation of fibrogenic stimuli and may represent injury processes. These generally occur in pathological conditions, such as pressure or volume overload [2] and diabetes mellitus [10]. Additionally, fibrosis in the subendocardial region is often observed in HF in endomyocardial biopsies [11]. In the progression of reactive fibrosis, ‘low-grade (smoldering) chronic inflammation’, mediated by macrophages around the perivascular spaces, might be involved [12,13,14]. These patterns usually coexist in most HF patients [15,16], thus it is practically difficult to distinguish between reactive and replacement fibrosis caused by small infarct lesions with low-flow ischemia in the advanced stages of myocardial diseases [6,7].

## 3. Cardiac ECM Characteristics and Homeostasis

**Figure 1:**
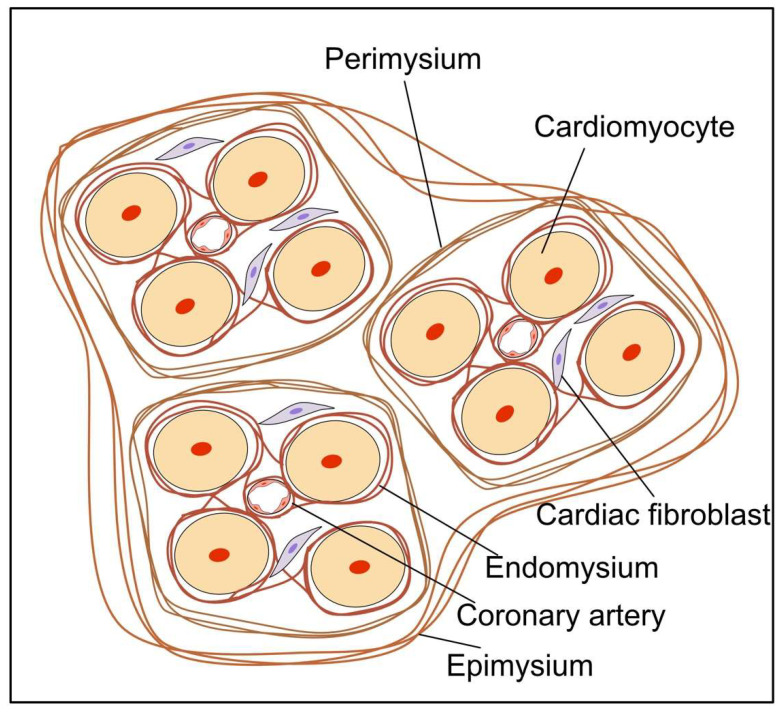
Schematic image of the cardiac interstitial collagen network. The endomysium surrounds and interconnects individual cardiomyocytes. The perimysial weave segregates cardiomyocytes into groups. The epimysium surrounds and clusters large numbers of myofibres.

Cardiomyocytes are surrounded by an interstitial ECM network composed mainly of fibrillar collagens [2,16]. The predominant components of the adult human heart ECM are collagen type I (85%) and collagen type III (11%) [16]. Although they are present in smaller amounts, type IV and V collagen are also observed in the heart [17,18]. The ECM is a highly durable, mechanically stable fiber-containing structure that preserves cardiac configuration and function and regulates cardiac diastolic and systolic operations by transmitting contractile forces from individual cardiomyocytes. The matrix consists of an endomysium, perimysium, and epimysium arranged around the myofibrillar bundles and coronary vasculature. The endomysial collagen network interconnects individual cardiomyocytes with Z-band-integrin connections and adjacent capillaries, preventing ventricular dilatation by maintaining cardiomyocyte alignment [19,20,21,22,23]. The perimysial collagen weave segregates cardiomyocytes into bundles to provide tensile strength [24]. The epimysium is a larger collagen weave surrounding several myofibers to preserve their orientation [24] (Figure 1). In addition to collagens, the cardiac ECM contains various glycoproteins, glycosaminoglycans, proteoglycans, and soluble factors [25]. Under pathological conditions, excess ECM deposition exacerbates tissue stiffness and can interrupt cell–cell connections, preventing adequate contractions and signal transduction. Collectively, ECM components are affected by aging [26] and pathophysiological conditions including hypertensive heart disease, heart failure with preserved ejection fraction (HFpEF), and ischemic heart disease [16,27,28,29].

CFs and activated CFs are the main cell types responsible for maintaining ECM homeostasis. CFs synthesize collagen as a soluble procollagen with N- and C-terminal propeptide regions that prevent insoluble deposition [30]. Sequential processing steps are required in the extracellular space for procollagen to become a mature fibril collagen. A disintegrin and metalloproteinase with thrombospondin motifs (ADAMTS) 2, 3, and 14 cleave the N-terminal [31] (also referred to as procollagen amino-terminal proteinases (PNPs)), whereas bone morphogenic protein (BMP)-1 is the protease responsible for cleaving the C-terminal [32,33] (also referred to as procollagen carboxy-terminal proteinases (PCPs)). Additionally, PCP enhancer (PCPE)-1 and 2, enhancers of BMP-1 activity, facilitate C-terminus cleavage [34]. Collagens are further stabilized by cross-linking that occurs by lysyl oxidase (LOX)-mediated aldehyde formation in lysine or hydroxylysine residues [35] and transglutaminase 2 by the formation of ε (γ-glutamyl) lysine cross-links [36]. Collagen cross-linking via these mechanisms promotes myocardial stiffness [37,38]. CFs are also the predominant source of matrix metalloproteinases (MMPs), which are calcium-dependent zinc-containing endopeptidases responsible for matrix protein degradation. MMPs are involved in collagen deposition and pro-fibrotic signaling and are important for cardiovascular diseases [39]. MMPs are tightly regulated by an endogenous group of inhibitors known as tissue inhibitors of metalloproteinases (TIMPs) [40]. All four known TIMPs are expressed in the heart, predominantly produced by CFs but also a variety of other cell types. TIMP expression is correlated with cardiac diseases [40,41,42,43,44].

## 4. Cardiac Fibroblasts

CFs are an essential cell type, mainly derived from the proepicardium, endothelial cells, and neural crest cells, and reside within the myocardial and valve interstitium, epicardial, and perivascular regions [45,46]. A subset of CFs in the interventricular septum and valves are derived from the endocardium through endothelial-to-mesenchymal transition (EndoMT) [47]. CFs are essential for normal cardiac function [2,48] and mediate various physiological forces, including mechanical and electrical stimuli. CFs produce paracrine factors that significantly change the electrophysiological activity in rat cardiomyocytes [49]. CFs can interact with cardiomyocytes through gap-junctional proteins, such as connexins (e.g., Cx40, Cx43, and Cx45) [50,51,52,53]. Additionally, CFs are widely accepted as the main regulator of the heart’s response to various pathological injuries, such as ECM production, matrix degradation, inflammatory cell recruitment, and scar formation [27]. After an acute myocardial injury, the expression of various proinflammatory cytokines is upregulated in the initial inflammatory response in CFs, resulting in subsequent inflammatory cell infiltration and cytokine expression in the heart [54]. In this setting, mechanical stress and inflammation stimulate CF activation, which shows morphological characteristics of both fibroblasts and smooth muscle cells with the expression of α smooth muscle actin (αSMA) [55,56]. Activated CFs secrete elevated levels of collagen and other ECM proteins. This reaction maintains the heart’s structural integrity and pressure-generating capacity. In the advanced phases of fibrotic scar formation, the tensile strength of collagen increases within the injury site [2]. Excess deposition of ECM proteins by CFs reduces ventricular compliance and worsens HF. In addition, excess ECM deposition and fibroblast proliferation disturb the mechano-electric coupling of cardiomyocytes, thereby increasing the risk of arrhythmia and mortality [16]. Furthermore, inflammation and fibrosis within perivascular regions decrease the tissue availability of oxygen and nutrients and perpetuate the pathological response.

## 5. CF Activation and Activation Reversal

**Figure 2:**
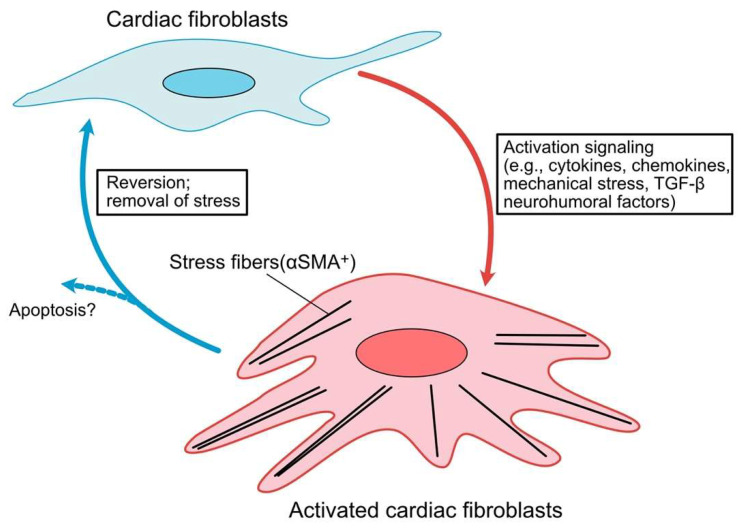
Progression and characterization of a cardiac fibroblast to myofibroblast conversion. In response to cardiac injury, cytokines, chemokines and neurohumoral factors, resident cardiac fibroblasts (CFs) become activated with increasing expression of α smooth muscle actin (α-SMA). These activated CFs may undergo apoptosis following the scar formation. Activated CFs are capable of de-differentiating upon removal of stress stimuli.

In response to cardiac injury, activated CFs release cytokines, chemokines, and neurohumoral factors [57,58]. Injured tissues secrete several inflammatory molecules, including cardiomyocytes, endothelial cells, fibroblasts, and inflammatory cells. They stimulate CF activation through diverse signaling pathways [48,59]. Pro-inflammatory cytokines (e.g., interleukin (IL)-1α, IL-1β, IL-6, and tumor necrosis factor (TNF)-α), chemokines (e.g., C-X-C motif ligand (CXCL) 1, 2, 5, and 8) [60], mechanical stress through integrins [59] and mechanosensitive ion channels [61] mediated by several kinase signaling pathways, including focal adhesion kinase (FAK) [62], Rho-associated protein kinase (ROCK) [63] and mitogen-activated protein kinase (MAPK) [64], growth factors, and neurohormonal factors have been shown to induce the activation of fibroblasts [29,65]. These activated CFs undergo apoptosis following scar formation [66]. However, recent studies using CFs derived from human HF patients indicate that inhibiting transforming growth factor (TGF)-β signaling reverts activated fibroblasts to resting fibroblasts, as determined by the reduction in αSMA expression [67]. Another study using an angiotensin II (Ang II) and phenylephrine infusion mouse model supported this observation. Two weeks after the cessation of drug infusion, gene expression reverted to resting fibroblast profiles [68]. Given recent findings that fibroblasts have plasticity in the gene expression profiles between resting and activated states and their significant heterogeneity revealed by single-cell RNA-seq analysis [69,70], there may be a threshold for whether activated fibroblasts undergo apoptosis or reversion to a resting state (Figure 2). Further studies are needed to answer these fundamental questions.

## 6. CF Markers

Specific CF and activated CF biomarkers are difficult to determine. Recent single-cell analysis in the adult human heart has revealed significant heterogeneity in CFs [71]. Collectively, in fibroblasts from different tissues, there is also significant heterogeneity in gene expression [72]. Thus, depending on the pathophysiological situation and anatomical site of the heart, it is necessary to combine several markers. The markers often used to identify fibroblasts are periostin, CD90 (or Thy1), discoidin domain receptor 2 (DDR2), platelet-derived growth factor receptor α (PDGFRα), transcription factor (Tcf) 21, fibroblast-specific protein (FSP) 1, stem cell antigen (Sca)-1, fibronectin, vimentin, and collagen types I and III. αSMA is the most commonly used marker for activated fibroblasts. Tenascin-C (TN-C) is highly expressed in activated CFs and is a potential marker for confirming their phenotype [6,7]. Although these are still under investigation, recent single-cell analyses have revealed several candidate markers that are expressed in cardiac valve interstitial fibroblasts, including *wif1* and the cartilage oligomeric matrix protein (COMP) [70]. Additionally, dermatopontin (Dpt)-CreERT2 mice efficiently mark universal fibroblasts [73] (Table 1).

## 7. Origin of CFs in the Development of Cardiac Disease

The embryonic proepicardium, which can give rise to various tissues, including pericytes, smooth muscle cells, cardiomyocytes, and endothelial cells [91], has been identified as the major cell source of CFs in numerous developmental models [45,46]. Endothelial cells are another source of CFs. Cardiac valves are composed of fibroblast-like valve interstitial cells (VICs) and valve endothelial cells (VECs) encompassing VICs. VICs are produced by the EndoMT of VECs [92,93,94]. Additionally, some CFs in the interventricular septum are derived from endothelial cells through EndoMT, as confirmed using genetic lineage tracing with Tie2-Cre mice [47]. Neural crest cells contribute to CFs within the atrium and VICs in semilunar valves [95,96,97]. Although the origin of fibroblasts in cardiac disease models has been controversial and is still under investigation, accumulating evidence suggests that the EndoMT, recruitment of circulating fibroblast progenitors, and expansion of resident fibroblasts may contribute to cardiac fibrosis after cardiac injury [68,98,99,100,101,102].

## 8. Signaling Pathways in Cardiac Fibrosis

Numerous signaling pathways have been implicated in CF activation and pathological remodeling progression. Modulating these signaling pathways as novel therapeutic targets is of great interest. Therefore, we summarized the mediators and signaling pathways that influence CF function after cardiac injury.

### 8.1. TGF-β Signaling

**Figure 3:**
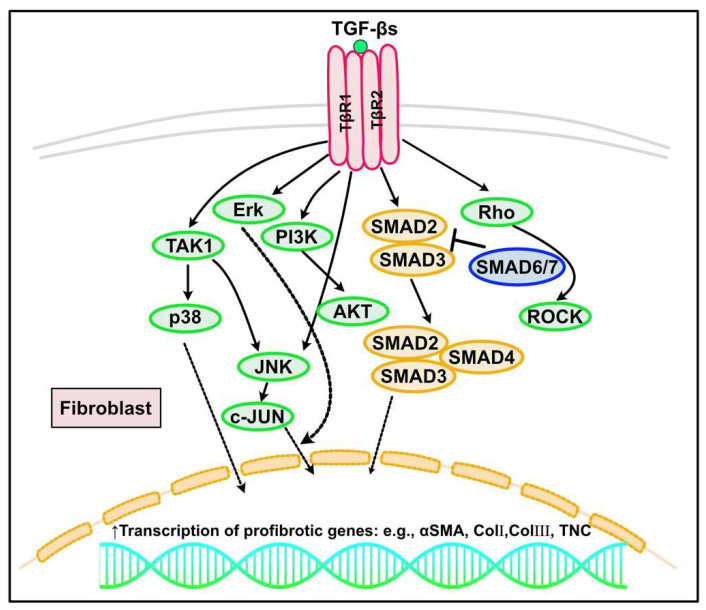
TGF-β signaling in cardiac fibrosis. TGF-β could induce the signal transduction via the canonical (SMAD-dependent) and non-canonical (SMAD-independent) pathways. In the canonical pathway, TGF-β1 binds to and causes heterodimerization of TGF-β receptor type 1 (TβRI, also known as activin-like kinase (ALK) 5) and the type II receptor (TβRII), leading to the phosphorylation of SMAD2/SMAD3, which subsequently form a complex with SMAD4 and translocate into the nucleus, acting as a transcriptional factor to regulate the fibrotic gene expression (e.g., αSMA, collagen I, III or TNC). SMAD6/7 are inhibitory SMADs to inhibit transcription of SMAD2 and SMAD3. In canonical pathways, TGF-β1 can also induce SMAD-independent noncanonical signaling that involves several mitogen-activated protein kinases, including extracellular signal-regulated kinase (Erk), c-Jun-N-terminal kinase (JNK), TGF-β-activated kinase 1 (TAK1), Rho family of small GTPase, and p38 MAPK pathways.

TGF-β is one of the most vigorously researched fibrotic factors. It is a pleiotropic peptide with diverse effects. Its effects on cellular behavior are dependent on cell type and environmental and cellular conditions, and are regulated in a highly context-dependent manner [103]. TGF-β mediates various biological processes, such as embryonic development, tumor growth, cell proliferation, and apoptosis [104,105,106]. TGF-β is also a central player in hypertrophic and fibrotic remodeling of the heart, mediating cardiomyocyte growth, CF activation, inflammation, and ECM deposition [107,108,109]. TGF-β includes three isoforms (TGF-β1, TGF-β2, and TGF-β3) in mammals, encoded by three different genes [104,110]. Among the three isoforms, TGF-β1 is predominant. It is crucial in pathological fibrosis and is produced by various cells, including immune cells, endothelial cells, cardiomyocytes, and activated fibroblasts [111,112]. TGF-β1 is initially secreted as an inactive complex with latent TGF-β-binding proteins and TGF-β pro-peptides. This complex is cleaved and activated during an integrin-mediated process [113,114,115]. TGF-β can induce signal transduction via canonical (SMAD-dependent) and non-canonical (SMAD-independent) pathways. In the canonical pathway, TGF-β1 binds to and causes heterodimerization of TGF-β receptor type 1 (TβRI, also known as activin-like kinase (ALK) 5) and type II (TβRII), leading to SMAD2 and SMAD3 phosphorylation. Consequently, a complex with SMAD4 forms and translocates into the nucleus, acting as a transcriptional factor to regulate the expression of target genes [28,48,59,116]. Intriguingly, recent reports have suggested the distinct roles of SMAD2 and SMAD3 in mediating TGF-β signaling [117,118,119]. SMAD6 and SMAD7 are inhibitory SMADs. They can interact with TβRI and competitively inhibit SMAD2 and SMAD3 [116,120]. In addition to the SMAD-dependent canonical pathways, TGF-β1 can induce SMAD-independent non-canonical signaling that involves several mitogen-activated protein kinases, including extracellular signal-regulated kinases (ERKs), c-Jun N-terminal kinases (JNKs), TGF-β-activated kinase 1 (TAK1), Rho family of small GTPases, and p38 MAPK pathways [121,122]. In fibrosis regulation, TGF-β can transform fibroblasts into activated CFs and promote ECM synthesis and deposition [104], which involves SMAD3 signaling [123,124,125,126] (Figure 3). TGF-β also inhibits ECM degradation by regulating plasminogen activator inhibitor (PAI)-1 and TIMP expression levels [127]. Conversely, TGF-β can induce MMPs (e.g., MMP1, 2, and 3), suggesting that over-activation of TGF-β signaling may lead to aortic wall vulnerability in Marfan syndrome [128]. Additionally, non-canonical TGF-β signaling can induce fibrosis [121,129]. In human activated CFs, RNA-binding proteins, such as pumilio RNA binding family member 2 (PUM2) and KH domain-containing RNA binding (QKI), work as hub proteins of the canonical TGF-β1–SMAD and TGF-β1–MAPK pathway, and the non-canonical IL-11-mediated pathway, which regulates fibrogenic gene expression [130]. Vast cumulative evidence points to the role of non-coding RNAs and microRNAs in cardiac fibrosis [131,132,133,134,135]. Metabolic dysregulation, including glucose metabolism, is also involved in fibrosis progression through the regulation of TGF-β-mediated hypoxia-inducible factor (HIF)-1α and/or the renin-angiotensin system [136,137,138,139,140].

### 8.2. Renin-Angiotensin-Aldosterone System

**Figure 4:**
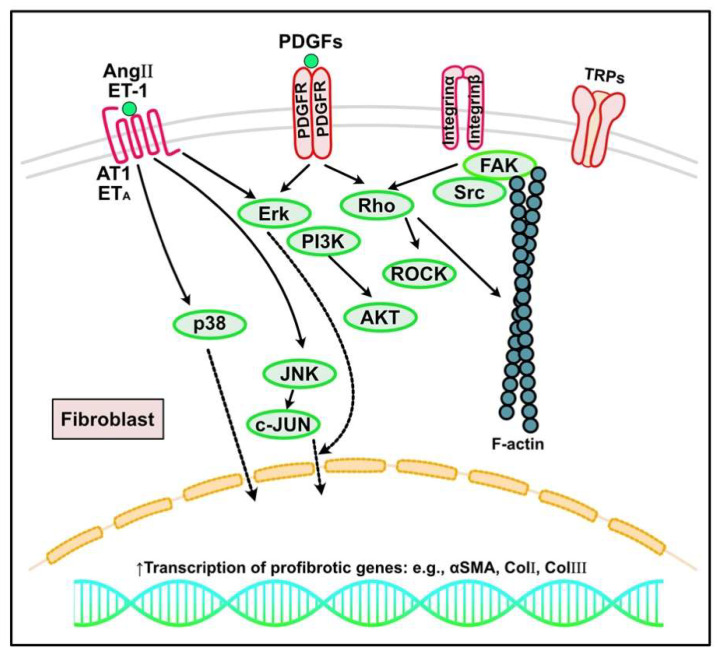
Other signaling pathways regulating cardiac fibrosis. In response to increased mechanical stress and cardiac injury, inflammatory signaling, growth factors (e.g., platelet-derived growth factors (PDGFs)), neurohumoral pathways (e.g., angiotensin (Ang) II, endothelin (ET)-1), and mechanosensitive pathways mediated by integrins and ion channels such as transient receptor potential cation channels (TRPs) can activate fibroblasts into myofibroblasts, leading to excess extracellular matrix protein deposition and cardiac fibrosis. AT1, angiotensin type 1 receptor; PDGFR, PDGF receptor; ERK, extracellular signal regulated kinase; PI3K, phosphoinositide 3-kinase; JNK, c-JUN N-terminal kinase; αSMA, α-smooth muscle actin; ROCK, Rho-associated protein kinases; FAK, focal adhesion kinase.

The renin-angiotensin-aldosterone system, in which Ang II is considered the most predominant isoform, promotes many pathophysiological functions, including cardiac fibrosis [141,142,143]. Systemically or locally produced Ang II acts through two specific receptors: angiotensin type (AT) 1 and AT2. Ang II through AT1 is involved in various biological processes, including CF proliferation, migration, and CF activation, with the induction of ECM protein synthesis and apoptosis [2]. In contrast, AT2 has a cardioprotective role, acting as a negative regulator of Ang II-mediated fibrogenic responses. AT2 inhibits AT1 action by suppressing CF proliferation and matrix synthesis [144]. The effects of Ang II through AT1 on CF activation are mediated through the activation of p38 MAPK, protein kinase C (PKC), and ERK cascades [145,146] (Figure 4). Ang II also interacts with TGF-β signaling in cardiomyocytes and CFs to induce cardiac hypertrophy and fibrosis. Various mediators regulate CF responses to Ang II through AT receptor expression. For instance, pro-inflammatory mediators (e.g., NF-κβ, IL-1β, IL-6, and TNF-α) make fibroblasts more responsive to Ang II by inducing AT1 synthesis [2,147].

### 8.3. Endothelin (ET)

ET was first identified as a potent vasoconstrictor peptide. It is now widely accepted as a multifunctional peptide involved in development, tumor growth, immune regulation, and cardiac fibrosis [148,149,150,151,152,153,154,155,156]. ET-1, the predominant isoform in humans, is thought to be secreted mainly by endothelial cells, but can also be produced by every cell type [157]. G protein-coupled receptors (GPCRs) ET_A_ and ET_B_ are two recognized ET-1 receptors. Although ET-1 acts mainly through ET_A_ to promote vasoconstriction, inflammation, and cell proliferation, the ET_B_ receptor is considered a physiological antagonist [157]. ET-1 exerts fibrogenic effects, acting as a downstream molecule of cytokines and neurohumoral mediators, thus linking inflammation and cardiac fibrosis [157,158] (Figure 4). Ang II and TGF-β induce ET-1 expression [159,160] and ET-1 upregulation is consistently confirmed in many fibrosis-associated cardiac pathologies, including MI, HF, and hypertensive heart disease [157,161]. Both genetic models and pharmacologic inhibition studies suggest the fibrogenic effects of ET-1 in myocardial disease. Cardiac ET-1 overexpression in mice induces myocardial fibrosis associated with both systolic and diastolic dysfunction [162]. Moreover, endothelium-specific loss of ET-1 attenuated fibrosis in Ang II-infused mice [163]. ET-1 inhibition improved cardiac fibrosis [164]. The anti-fibrotic effects of the ET-1 blockade may have therapeutic implications. ET_A_ and dual ET_A_/ET_B_ antagonists reduce myocardial remodeling by suppressing collagen deposition and attenuating cardiac fibrosis in animal models of aldosterone treatment that recapitulate the features of human HFpEF [165,166]. Although its effectiveness in animal experiments has been shown, clinical trials using ET_A_ antagonists have not been beneficial for patients with heart failure with reduced ejection fraction (HFrEF) and HFpEF [157].

### 8.4. Platelet-Derived Growth Factors (PDGF)

PDGFs have many pathophysiological roles in embryonic development, tumor progression, vascular diseases, and fibrosis. PDGFs form homo- or heterodimers and act through two receptor tyrosine kinases (PDGFR-α and PDGFR-β). They have common domain structures, including five extracellular immunoglobulin loops and a split intracellular tyrosine kinase domain. In fibrogenic conditions, PDGF signaling, which in part interacts with TGF-β signaling, causes cell proliferation with an activated phenotype, resulting in excessive ECM production and deposition [167] (Figure 4). PDGF-A, PDGF-C, and PDGF-D are implicated as potential fibrogenic PDGFs in the myocardium through direct actions and, in part, through TGF-β [168,169]. PDGF-AA stimulates CF proliferation in vitro [170]. PDGF-A or PDGF-D overexpression can cause cardiac fibrosis due to excess fibroblast activation [171,172]. Although controversial, PDGF-B is also a potent fibrogenic PDGF [172,173]. PDGFR-α activation is consistently involved in myocardial fibrosis. Treatment with a neutralizing antibody against PDGFR-α and PDGFR-β attenuated collagen deposition [173]. Additionally, a broader PDGFR blockade through the kinase inhibitor imatinib reduces cardiac fibrosis in mouse myocarditis, MI, and isoproterenol infusion models [174,175,176]. PDGFR-β activation potentially occurs through integrin β1 and small proline-rich repeat 3 to augment fibroblast proliferation and matrix synthesis in a cardiac pressure overload mouse model [177]. PDGFR-β signaling neutralization in infarcted hearts has reduced collagen deposition with significantly increased microvascular density and reduced vascular mural cell-coated vasculature, suggesting the importance of PDGFR-β actions on vascular mural cell recruitment and differentiation [167,169,178]. PDGFs have also been shown to be involved in the cardiac fibrotic response in an Ang II-treated mouse model [179]. Ang II-induced cardiac fibrosis is reduced in Krüppel-like zinc-finger transcription factor (*klf*) *5* heterozygous knockout mice, correlating with reduced cardiac expression of PDGF-A with direct binding of KLF5 to the PDGF-A promoter region [180]. Together, these results implicate PDGFR-α stimulation as a predominant fibrogenic process in the heart, whereas PDGFR-β stimulation also affects vascular mural cells.

### 8.5. Wnt Signaling

**Figure 5:**
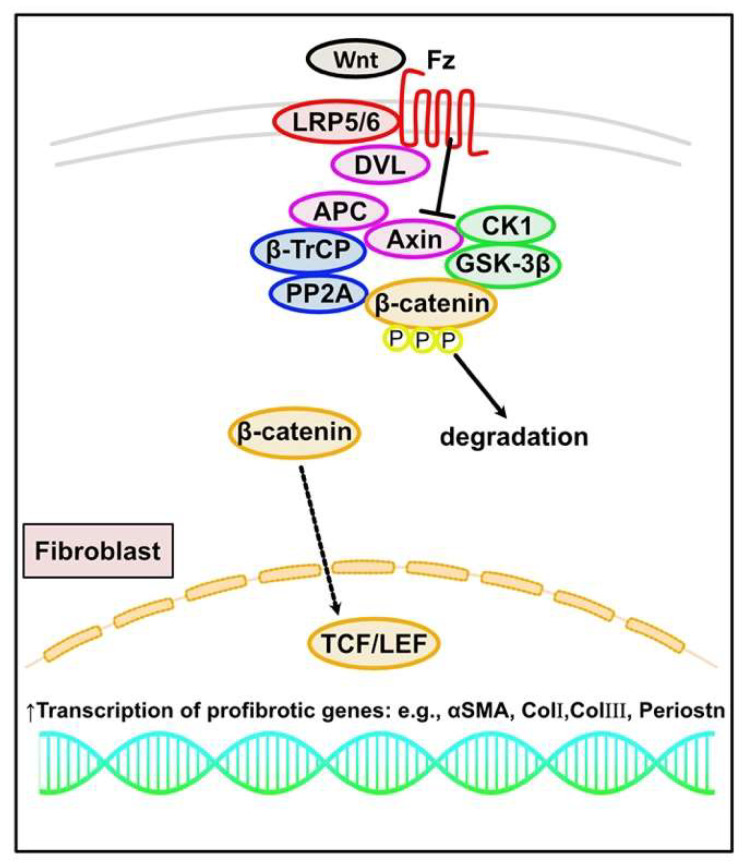
Canonical WNT/β-catenin signaling in cardiac fibrosis. In the absence of Wnt ligands, cytosolic β-catenin is degraded by the destruction complex, which includes Axin and adenomatous polyposis coli (APC), glycogen synthase kinase (GSK)-3β and casein kinase (CK)1, protein phosphatase 2A (PP2A), and β-transducin repeat-containing protein (β-TrCP). After a Wnt ligand binds to the receptor Frizzled (Fz) and the receptor-related protein 5 or 6 (LRP5/6) coreceptor, the Wnt–Fz–LRP5/6 complex recruits Disheveled (DVL) and Axin through the intracellular domains of Fz and LRP5/6, resulting in β-catenin stabilization. The increased nuclear levels of β-catenin promote interaction with T cell factor/lymphoid enhancer factor (TCF/LEF) transcription factor to regulate Wnt-responsive fibrotic genes.

The Wnt signaling pathway has diverse roles in many biological processes, including carcinogenesis, embryonic development, immune maintenance, and fibrosis [181,182,183]. Several reports have indicated essential roles for Wnt signaling in cardiac fibrosis progression, mainly through the TGF-β pathway. The canonical Wnt/β-catenin pathway is predominantly involved in cardiac fibrosis progression, interacting with SMAD-dependent canonical TGF-β signaling [184,185]. In the absence of Wnt ligands, cytosolic β-catenin is degraded by the destruction complex, which includes tumor suppressors Axin, adenomatous polyposis coli (APC), the serine/threonine kinases, glycogen synthase kinase (GSK)-3β, casein kinase (CK) 1, protein phosphatase 2A (PP2A), and the E3-ubiquitin ligase β-transducin repeat-containing protein (β-TrCP) [186]. After a Wnt ligand binds to the seven-pass transmembrane receptor Frizzled (Fz) and the single-pass low-density lipoprotein receptor-related protein 5 or 6 (LRP5/6) coreceptor, the Wnt–Fz–LRP5/6 complex recruits Dishevelled (DVL) and Axin through the intracellular domains of Fz and LRP5/6, resulting in β-catenin phosphorylation inhibition and β-catenin stabilization. Increased cytoplasmic and nuclear β-catenin levels promote interaction with the T cell factor/lymphoid enhancer factor (TCF/LEF) to regulate Wnt-responsive genes [187]. In CFs from MI mice and human, phosphorylated GSK-3β negatively regulates TGF-β signaling by directly interacting with SMAD3 and through β-catenin signaling. Moreover, GSK-3β deletion or inhibition in in vivo models leads to hyperactivation of TGF-β-SMAD3 signaling and cardiac fibrosis [188,189]. Secreted Fz-related proteins (sFRPs), which are endogenous modulators of Wnt signaling, have emerged as key regulators of the fibrotic response. sFRP1 inhibits Wnt ligands. sFRP1 null mice show cardiac dilation with increased expression of canonical Wnts, β-catenin, and Wnt target genes, such as *Lef1* and *Wisp1*, leading to increased α-SMA expression and collagen production [190,191] (Figure 5). sFRP2 is a Wnt signaling inhibitor. sFRP2 null mice exhibit reduced collagen deposition and improved cardiac function after MI [192]. sFRP2 binds BMP1 through its Fz domain, enhancing the interaction between BMP1 and procollagen, thereby promoting procollagen processing and collagen deposition in the ECM [191,193]. A pressure overload model with trans-aortic constriction in mice observed increased β-catenin signaling in CFs. Fibroblast-specific loss of β-catenin led to improved cardiac function with reduced interstitial fibrosis and decreased ECM expression by directly regulating the expression of ECM-related genes, such as *Col3a1* and *Postn* [194]. In contrast, injecting the sFRP2 protein into the infarcted left ventricle in a rat MI model inhibited cardiac fibrosis and improved cardiac function [193]. These controversial results may be caused by the use of different animal models and sFRP2 concentrations. Additional studies are needed to elucidate the action mechanisms of sFRP2 in cardiac fibrosis.

## 9. Matricellular Proteins That Regulate Cardiac Fibrosis

### 9.1. Tenascin-C

Matricellular proteins are a family of specialized ECM molecules that are upregulated at high levels during tissue remodeling and have many biological roles [195,196]. TN-C is a typical matricellular protein and large ECM glycoprotein that is dramatically upregulated during development and in pathological tissues and interacts with other biological signaling pathways. In the heart, TN-C is expressed in a spatiotemporally limited fashion during embryonic development, although TN-C knockout or overexpressing mice exhibit grossly normal heart morphogenesis [197]. TN-C is sparsely detected in normal mice and human adult hearts but is re-upregulated around injured tissues under pathological conditions, including MI, myocarditis, DCM, Kawasaki disease, and cardiac fibrosis [6,7,198]. In the Ang II infusion heart model, TN-C was produced by perivascular interstitial cells and deposited at fibrotic lesions around the macrophage infiltration site [179]. The induction of TN-C by ANG II is partially mediated by the ET1/ETA pathway, which could be suppressed by atrial natriuretic peptides [199]. Upregulated TN-C around the injured area stimulates macrophages via the integrin αvβ3/FAK/Src/nuclear factor-κB axis, facilitating the expression of proinflammatory cytokines, such as IL-6, which stimulate fibroblasts to synthesize excess collagen, leading to fibrosis. Additionally, proinflammatory cytokines produced by macrophages stimulate TN-C synthesis in CFs, leading to additional fibrosis in a positive feedback loop [200]. TN-C may also promote fibrosis by stimulating fibroblasts via integrin αvβ1/TGF-β/SMAD2 [201] and PDGF-AB/PDGFR [179]. TN-C deletion attenuates inflammation and fibrosis [6,7]. Similar TN-C proinflammatory and profibrotic roles have been reported in the transverse aortic constriction (TAC) model [202]. Locally produced TN-Cs may amplify monocyte/macrophage recruitment by increasing RhoA/ROCK signaling-dependent motility, thereby promoting a shift toward an inflammatory phenotype and upregulating chemokine expression in CFs. Similar to the Ang II-induced model, TN-C deletion attenuated inflammatory and fibrotic changes as well as hypertrophy and contractile dysfunction in TAC heart models [203,204].

### 9.2. Connective Tissue Growth Factor (CTGF)

CTGF, also known as CCN2, is a matricellular protein expressed in CFs and cardiomyocytes regulating diverse cellular functions, including cell adhesion, matrix production, structural remodeling, angiogenesis, and cell proliferation and differentiation. Mechanical stresses, numerous cytokines, neurohormonal factors, and growth factors, including TGF-β, regulate CTGF gene expression. CTGF mediates the downstream pro-fibrotic actions of TGF-β in the heart [205,206]. Both TGF-β and CTGF were expressed in CFs in MI models [190]. Increased CTGF causes ECM protein upregulation, including fibronectin and collagen I and III, following MI in an animal model [207]. CTGF is strongly upregulated in human HF and animal models associated with cardiac fibrosis [208,209,210]. Although CTGF is considered a pro-fibrotic factor, its role in cardiac diseases is controversial [211,212]. Further studies are needed to dissect these points for clinical applications.

### 9.3. Periostin, Osteopontin (OPN), and Secreted Protein Acidic and Rich in Cysteine (SPARC)

Periostin is also involved in cardiac fibrosis [213,214]. It is produced in activated fibroblasts through mechanical stress, chemokines, changes in matrix composition, and the canonical TGF-β pathway [215,216,217]. LOX and TN-C-associated periostin acts through integrin αvβ1, β3, and β5 to activate p38 [218], FAK, and PI3K/AKT [219,220], which control fibrogenic gene expression, resulting in cardiac fibrosis [221,222].

OPN, a glycoprotein with an arginine-glycine-aspartic acid (RGD) sequence, interacts with integrins (αvβ3) and the CD44 receptor in an RGD-dependent manner [2,223]. OPN mediates diverse biological functions, including cell adhesion, chemotaxis, and signaling. OPN may regulate TGF-β, MMPs, and LOX to exert cardiac fibrogenic effects [214,224,225,226,227].

The matricellular protein SPARC regulates post-synthetic collagen processing to facilitate the formation and assembly of mature cross-linked, insoluble structural collagen fibrils [2]. The absence of SPARC causes alterations in ECM fibrillar collagen and cardiac function [228]. SPARC also induces TGF-β signaling and activates ADAMTS1. ADAMTS1 promotes collagen fiber assembly, stabilization, and release in the heart, leading to cardiac fibrosis [229].

## 10. Cardiac Lymphatics

The heart has an extensive lymphatic vessel network comprising a heterogeneous origin and signaling that regulates its development [230,231,232,233,234,235]. In a mouse MI model [236], cardiac lymphatic vessel impairment leads to interstitial fluid accumulation and myocardial fibrosis progression due to increased interstitial fluid pressure that might induce CF activation through mechanical stress within the ECM [237]. Additionally, lymphatic vessel dysfunction and lymphangiogenesis inhibition delay the resolution of inflammation [238]. Thus, stimulating cardiac lymphangiogenesis with growth factors (e.g., VEGF-C) in MI model mice reduces fibrosis and preserves cardiac function [231,232,237,239]. Cardiac edema induced by lymphatic ligation also increases collagen mRNA expression and deposition, enhancing cardiac fibrosis in rabbits. This confirms the importance of lymphatic vessels in cardiac fibrosis progression [240]. Recent reports suggest that lymphangiocrine signals from lymphatic vessels are another potential therapeutic target to promote cardiac growth and repair [241]. Thus, lymphatic vessels may regulate cardiac fibrosis through lymphangiocrine signaling.

## 11. Future Directions and Conclusions

Cardiac fibrosis influences HF progression and represents a major unmet public health requirement. Accumulating evidence points to the pathophysiologic heterogeneity of cardiac fibrosis and the complexity of CFs. Thus, it is very complicated to develop anti-fibrotic strategies for cardiac disease. Considering the complexity of CFs in health and disease, it is critical to identify the detailed relationships between distinct transcriptomic or proteomic profiles and functional properties. Collectively, it is important to investigate when and what cell types in cardiac fibrosis progression should be examined. Developing more specific in vivo approaches and identifying more specific targets is urgently required, and improving the understanding of cardiac fibrosis pathophysiology will lead to better-precision medicine-based treatment.

## Figures and Tables

**Table 1:** Marker proteins:DDR2, discoidin domain receptor 2; PDGFRα, platelet derived growth factor receptor α; TCF21, Transcription factor 21; FSP1, fibroblast specific protein 1; Sca-1, stem cell antigen-1; αSMA, α smooth muscle actin; TNC, tenascin-C. Modified from [48,55,73].

Markers/Mouse Lines	Functions	Expression in CFs	Expression in Other Cells in the Heart	References
Periostin	ECM protein	activated CFs	Epicardium	[55,68,73]
CD90	Cell adhesion and cell communication	Resting and activated CFs	Pericytes, VSMC, ECs, immune cells	[73,74]
DDR2	A membrane collagen-binding tyrosine kinase receptor	Resting CFs	Epicardium	[48,75,76]
PDGFRα	Tyrosine kinase receptor	CFs during development and after injury	Cardiac progenitor cells	[47,68,77,78]
TCF21	Involved in epithelial-mesenchymal transition	Resting CFs	Epicardium	[79,80]
FSP1	Calcium binding and promote filament depolymerization	Resting and activated CFs	Pericytes, VSMC, ECs, immune cells	[81,82]
Sca-1	Stem cell antigen	Resting and activated CFs	Cardiac progenitor cells	[83,84]
Fibronectin	ECM protein	Resting and activated CFs	ECs	[55,73,85]
Vimentin	Intermediate filament protein	Resting and activated CFs	Pericytes, VSMC, ECs	[86,87]
collagen types I and III	ECM protein	Resting and activated CFs	Pericytes, VSMC, ECs, cardiomyocytes	[55,88]
αSMA	Isoforms of actin filament	activated CFs	Pericytes, VSMC, cardiomyocytes, Epicardium	[2,29,89]
TNC	ECM protein	activated CFs	Pericytes, VSMC,	[6,7,90]
